# Supplementary material for: An Acid-Sensitive Bone Targeting Delivery System Carrying Acacetin Prevents Osteoporosis in Ovariectomized Mice
Source: Pharmaceuticals (Basel). 2022 Dec 20;16(1):2. doi: 10.3390/ph16010002 (PMC9867347; doi:10.3390/ph16010002)
Supplement: Supplementary file 1 [file pharmaceuticals-16-00002-s001.zip › pharmaceuticals-2074514-supplementary.docx]

**Supporting Information**

### An acid-sensitive bone targeting delivery system carrying acacetin prevents osteoporosis in ovariectomized mice

Xiaochen Sun^1#^, Chenyu Song^1#^, Chenxi Zhang^2#^, Chunlei Xing^2^, Juan Lv^2^, Huihui Bian^2^, Nanning Lv^3,4^, Dagui Chen^2^, Xin Dong^1*^, Mingming Liu^3,4*^, Li Su^2*^

^1^School of Medicine, Shanghai University, Shanghai, China

^2^Institute of Translational Medicine, Shanghai University, Shanghai, China

^3^Department of Orthopedics, Lianyungang Second People’s Hospital, Lianyungang, China;

^4^Lianyungang Clinical School, Xuzhou Medical University , Lianyungang, China

Correspondence: Li Su (suli1020@shu.edu.cn, Institute of Translational Medicine, Shanghai University, Shangda Road 99, Shanghai, 200444, China), Mingming Liu (drliumingming@163.com, Lianyungang Second People’s Hospital, East Hailian Road 41, Lianyungang, 222002, China) and Xin Dong (dongxin@shu.edu.cn, School of Medicine, Shanghai University, Shangda Road 99, Shanghai, 200444, China)


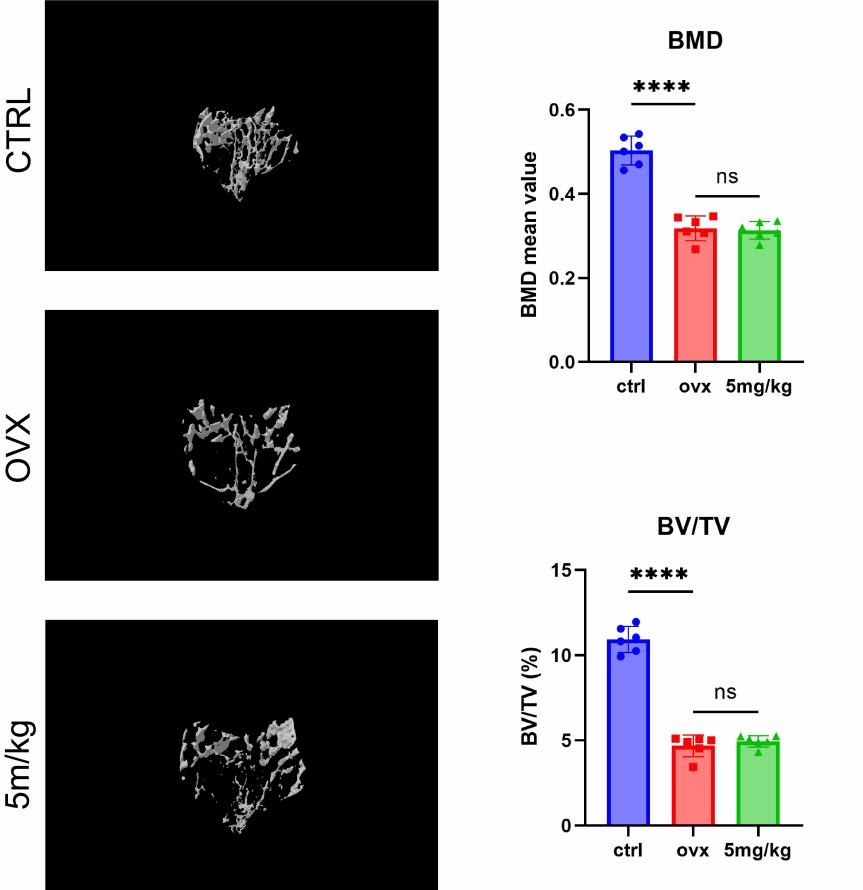


**Figure S1 . Treatment with 5mg/kg Acacetin has no effect on OVX-induced bone loss in mice.** One week after surgery of ovariectomy (OVX), mice were treated with Acacetin-EMCH-D6 or acacetin (i.p., 5mg/kg/3 days) for 5 weeks. Left tibia was subjected to the micro-CT scanning. Representative three dimensional reconstructed images were presented. Trabecular structural parameters of proximal tibia and vertebrae including BMD and BV/TV **** P<0.0001 versus the control group; n=6.

**1. Materials and instruments**

All amino acids were commercially available from GL Biochem (Shanghai) Ltd. Other reagents and solvents were brought from Acros, Sigmal-Aldrich, Alfa Aesar, Sinopharm Chemical Reagent Co., Ltd., Innochem Chemical Reagent). Dichloromethane (DCM) and THF were distilled over calcium hydride (CaH_2_) or NaH under argon atmosphere. All reactions vessels were oven-dried before use. Reactions were monitored by thin-layer chromatography (TLC) and visualized by UV analyzer (254 nm). ^1^H- and ^13^C-NMR spectrums were recorded on a Bruker 600 MHz instrument. Chemical shifts (*δ*) were reported relative to TMS (0 ppm) for ^1^H-NMR and ^13^C-NMR spectra. The coupling constants (J) were displayed in Hertz (Hz) and the splitting patterns were defined as follows: singlet (s); broad singlet (s, br); doublet (d); doublet of doublet (dd); triplet (t); quartet (q); multiplet (m). ESI-MS was measured with a Bruker Esquire-LC mass spectrometer. High resolution mass spectra were measured on a Waters Xevo G2 QTOF mass spectrometer.

2. Synthesis and chemical characterization

*(E)-N'-(5,7-dihydroxy-2-(4-methoxyphenyl)-4H-chromen-4-ylidene)-6-(2,5-dioxo-2,5-dihydro-1H-pyrrol-1-yl)hexanehydrazide (3)*

To a solution of acacetin (1, 9.0 g, 31 mmol) and 6-(2,5-dioxo-2,5-dihydro-1H-pyrrol-1-yl)hexanehydrazide (2, 8.5 g, 38 mmol) in toluene was added catalytic *p*-toluenesulfonic acid. The reaction mixture was refluxed for 2 hours and then, the solvent was concentrated and the residue was purified by column chromatography (3:1-1:1, PE/EA) to afford 3 as a yellow powder (11.8 g, 76 %).

**^1^H-NMR (600 MHz, *d*-DMSO)**: δ 8.02 (d, *J*=12 Hz, 2H), 7.10 (d, *J*=6 Hz, 2H), 7.01 (s, 2H), 6.85 (s, 1H), 6.54 (d, *J*=6 Hz, 1H), 6.24 (d, *J*=6 Hz,1H), 3.86 (s, 3H), 3.38 (t, *J*=6 Hz, 2H), 2.22 (t, *J*=12 Hz, 2H), 1.55-1.48 (m, 4H), 1.23-1.20 (m, 2H);

**^13^C-NMR (600 MHz, *d*-DMSO)**: δ 182.22, 172.01, 171.53, 164.81, 163.72, 162.75, 161.86, 157.76, 134.92, 128.75, 123.30, 115.03, 104.17, 103.98, 99.40, 94.51, 56.01, 37.36, 32.91, 28.11, 26.03, 24.59；

**ESI-MS m/z:** calculated for C_26_H_25_N_3_O_7_ 491.16; found [M+H]^+^=492.25.

*Ac-Cys(Trt)-Asp(OtBu)_6_-resin (4)*

The 2-chlorotritylchloride resin (1 g, loading capacity=0.93 mmol/g, 1% DVB, 100-200 mesh) was swollen in DCM/DMF mixture solvent for 10 minutes. Fmoc-protected amino acid (3 equivalents), HOBt (3 equivalents) and DIC (9 equivalents) were pre- activated in DMF for 15 min before added into the peptide reactor. After 2 hours, the resin was washed (5×DMF, 5×DCM, 5×DMF). 20% piperidine/DMF solution was added to remove the Fmoc protection group. After all amino acids were coupled, Ac_2_O/pyridine mixed solution (1:1, v/v) was added and stirred for 20 min to afford *N*-terminal acetylated on-resin peptide 4.

*Ac-Cys-Asp_6_-OH (5)*

The cleavage cocktail (TFA/TIPs/water=95:2.5:2.5, *v/v/v*) was added to the resin at room temperature. After stirring for 2 hours, the cleavage cocktail was collected and concentrated. The chilled diethyl ether was added to the concentrated residue to precipitate the crude peptides. The peptide suspensions were centrifuged for 3 minutes at 3000 rpm and then the clear solution was decanted to afford crude peptide 5. The crude peptides were dissolved with MeCN/H_2_O and purified by semi-preparative RP-HPLC to afford 5 as a white powder (218 mg, 80%) after lyophilization.

**ESI-MS m/z:** calculated for C_29_H_39_N_7_O_21_S 853.19; found [M+H]^+^=854.40.

Acacetin-EMCH-D6

3 (200 mg, 0.4 mmol) and 5 (521 mg, 0.6 mmol) was dissolved in 10 mL sat. NaHCO_3_/MeCN solution (1:1, v/v), and the mixture was stirred for 30 min which was monitored by HPLC. Then, it was directly purified by semi-preparative RP-HPLC to afford Acacetin-EMCH-D6 as a white powder (381 mg, 71 %) after lyophilization.

**ESI-MS m/z:** calculated for C_55_H_64_N_10_O_28_S 1344.36; found [M+2H]^2+^=673.55, [M+H]^+^=1345.85.


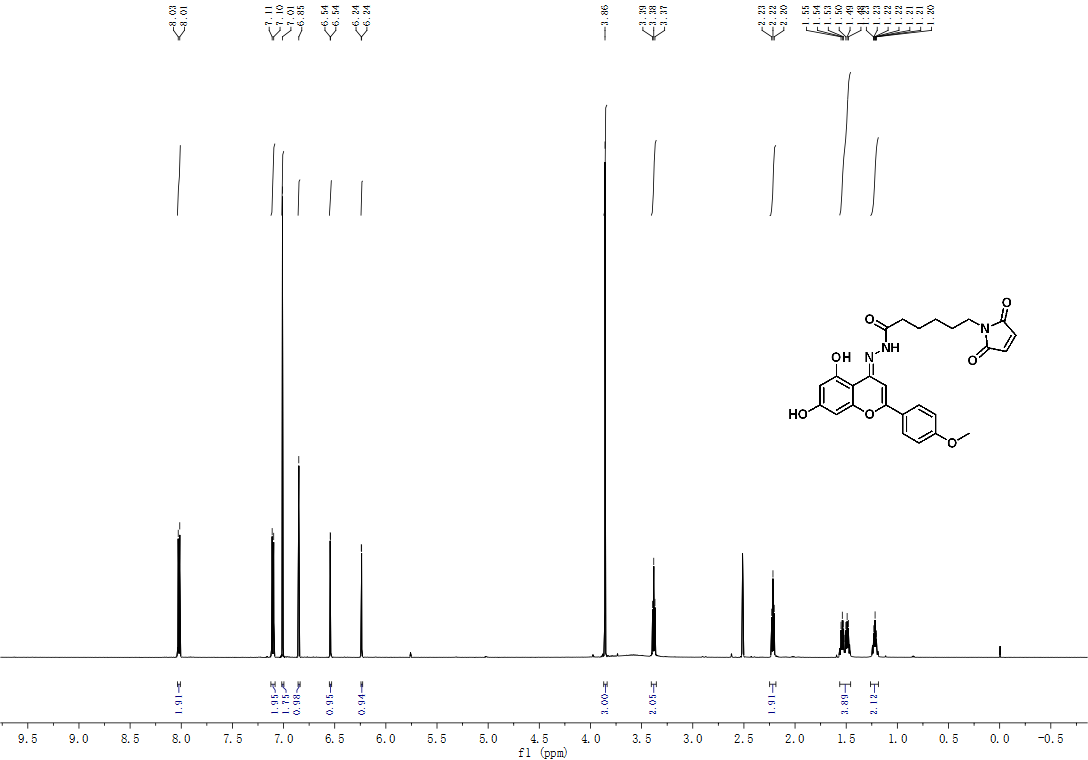


**Figure S2.** ^1^H-NMR data of 3.


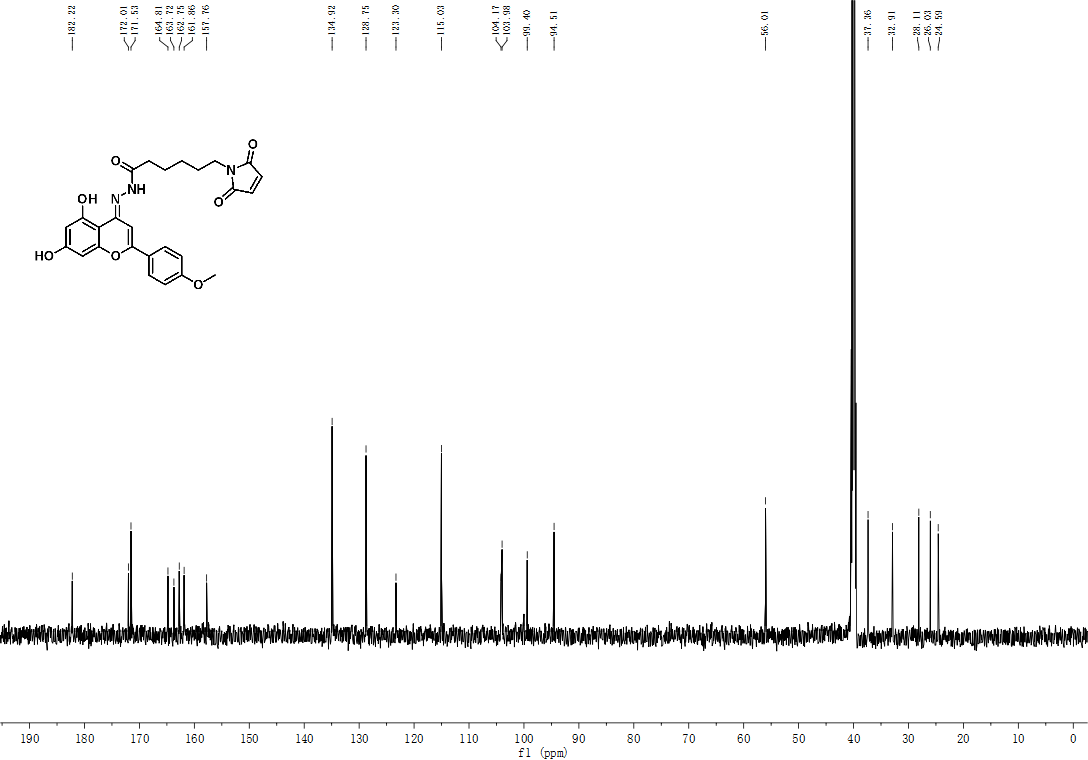


**Figure S3.** ^13^C-NMR data of 3.


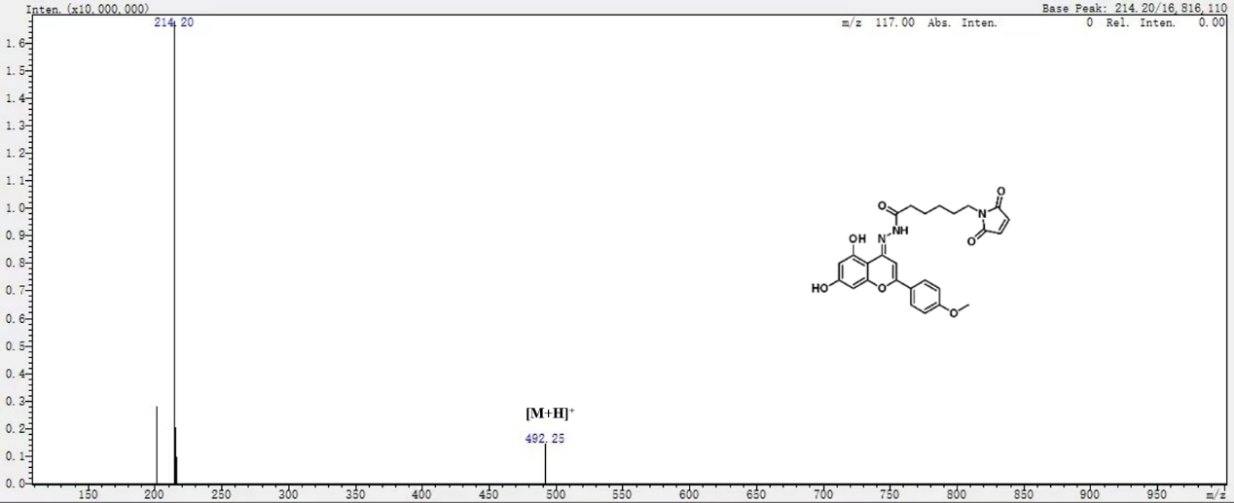


**Figure S4.** ESI-MS spectrum of 3 (calculated for C_26_H_25_N_3_O_7_ 491.16; found [M+H]^+^=492.25).


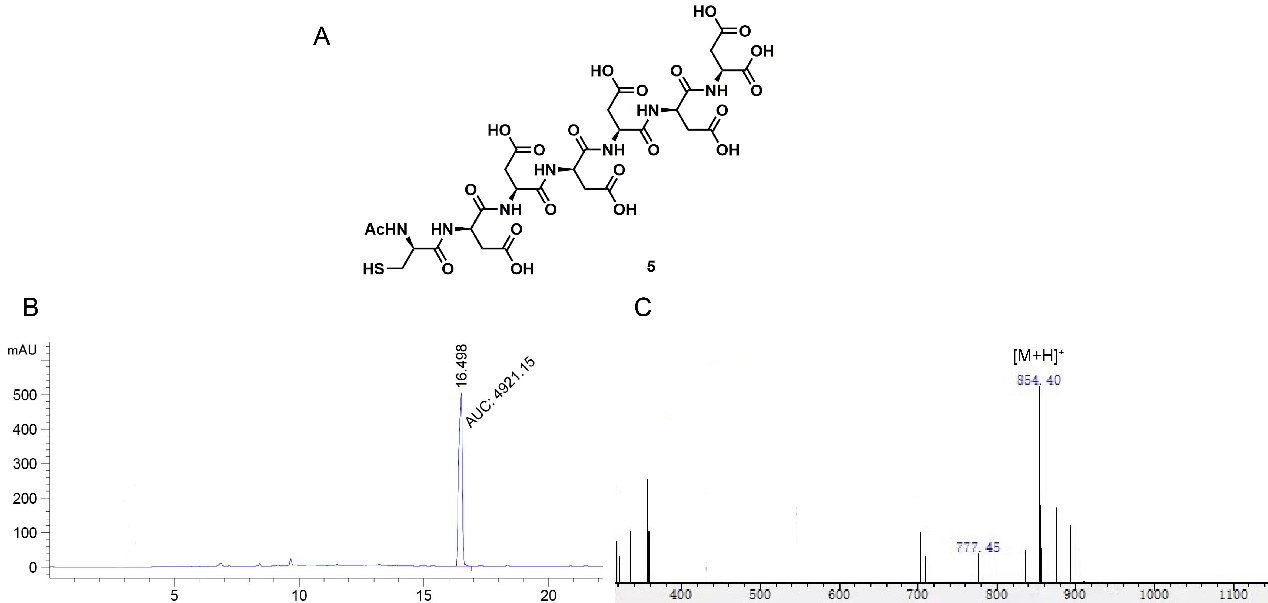


**Figure S5**. A) Structure of 5; B) HPLC trace of purified 5. Gradient: 90-0% of buffer B in 20 min with C_18_ column (5 µm, 2.5 mm×250 mm). C) HR-MS spectrum of 5 (calculated for C_29_H_39_N_7_O_21_S 853.19; found [M+H]^+^=854.40).


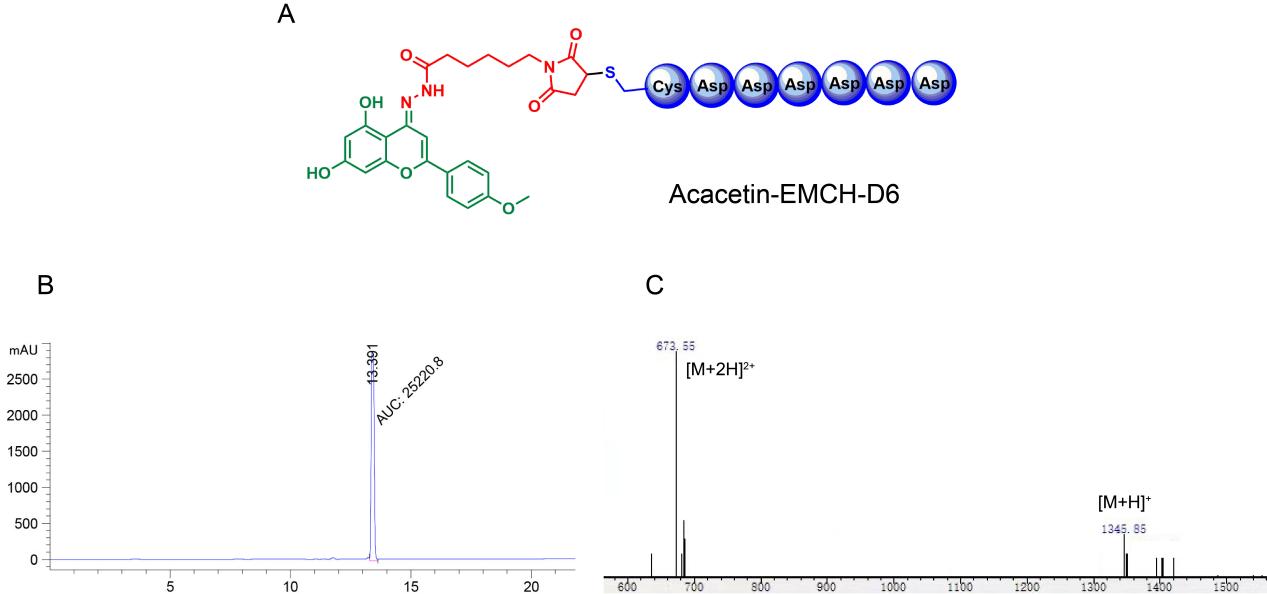


**Figure S6.** A) Structure of Acacetin-EMCH-D6; B) HPLC trace of purified Acacetin-6D. Gradient: 90-0% of buffer B in 20 min with C18 column (5 *µ*m, 2.5 mm×250 mm). C) HR-MS spectrum of Acacetin-EMCH-D6 (calculated for C_55_H_64_N_10_O_28_S 1344.36; found [M+2H]^2+^=673.55, [M+H]^+^=1345.85).


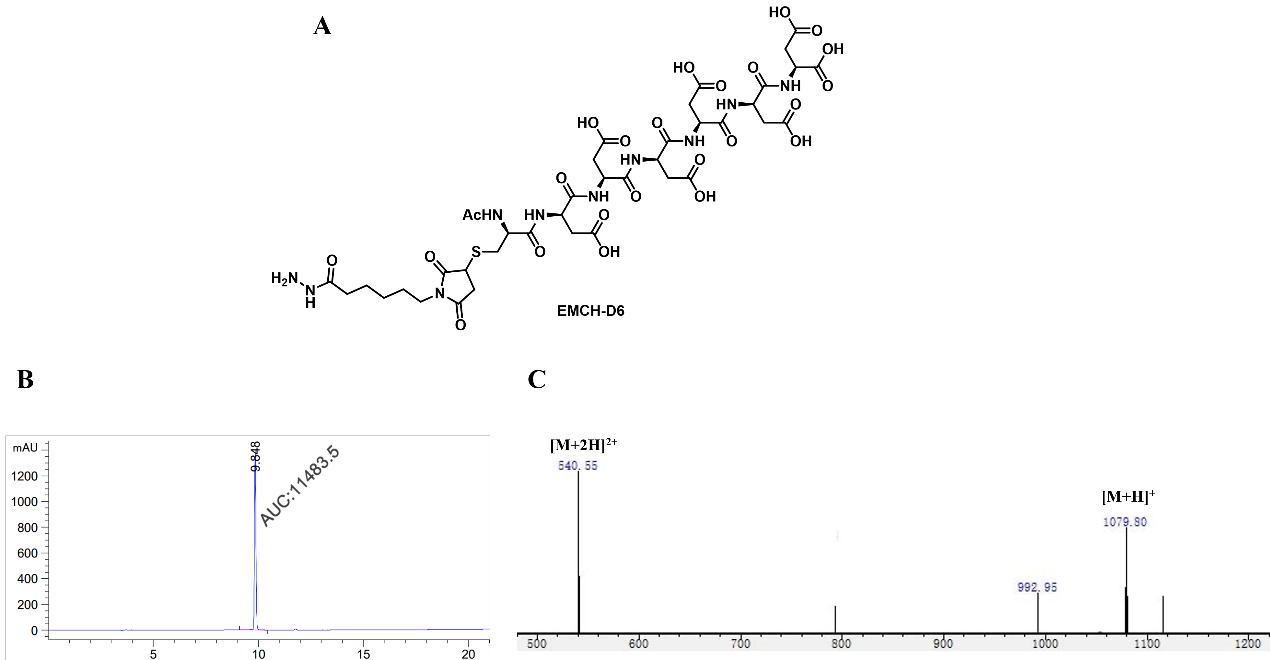


**Figure S7.** A) Structure of EMCH-D6; B) HPLC trace of purified EMCH-D6. Gradient: 90-0% of buffer B in 20 min with C18 column (5 *µ*m, 2.5 mm×250 mm). C) HR-MS spectrum of EMCH-D6 (calculated for C_39_H_54_N_10_O_24_S 1078.30; found [M+2H]^2+^=540.55, [M+H]^+^=1079.80).


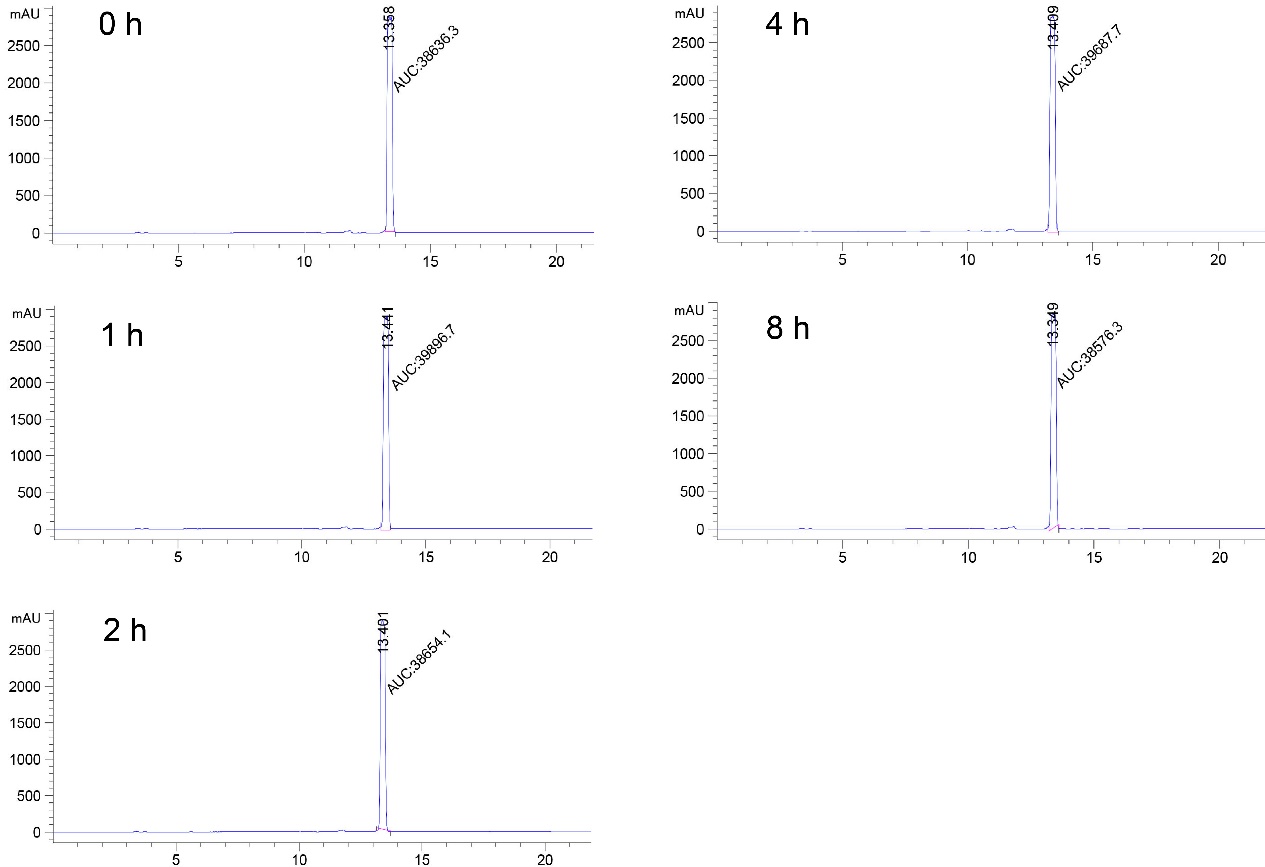


Figure S8. Chromatograms of chemical stability study of Acacetin-EMCH-D6 at pH=7.4 (37 °C).


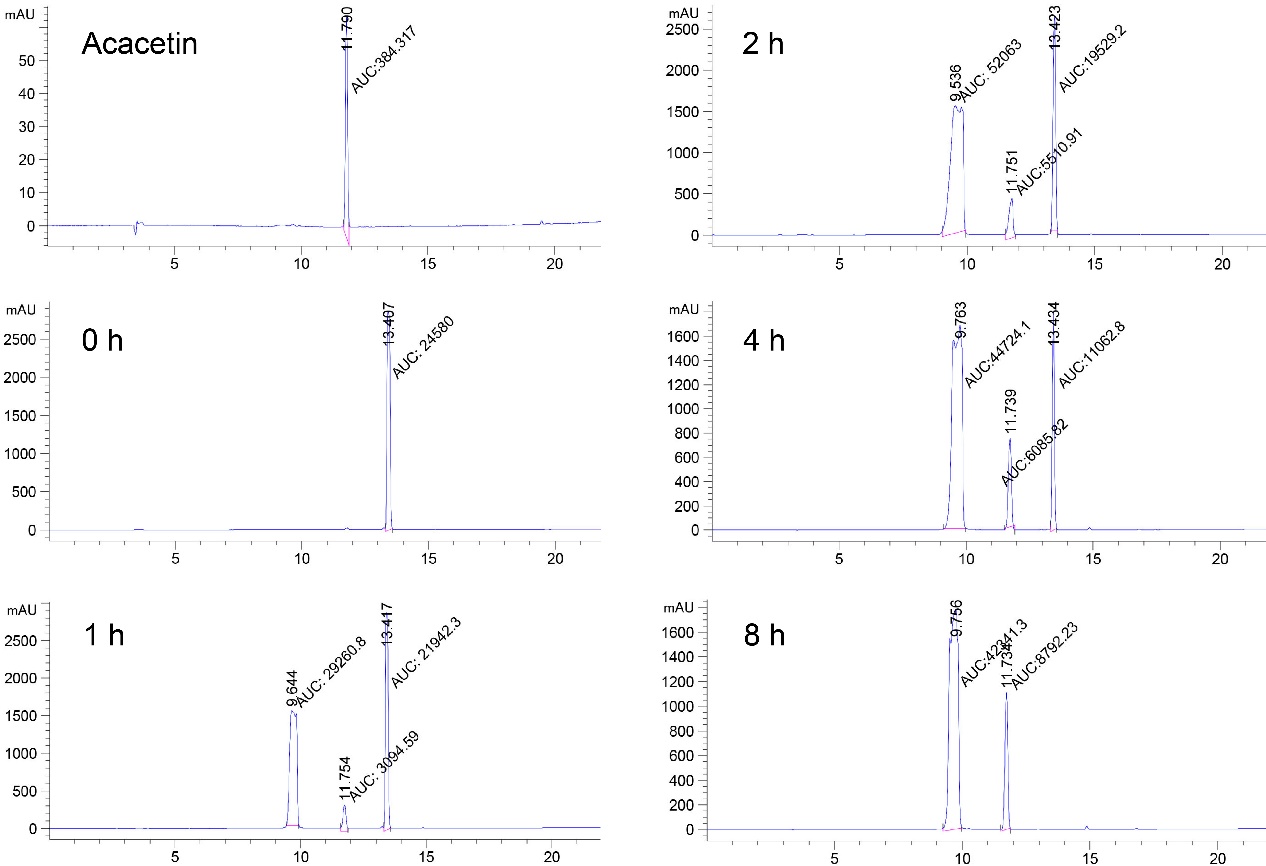


### Figure S9. Chromatograms of drug release study of Acacetin-EMCH-D6 at pH 5.5 (37 °C).


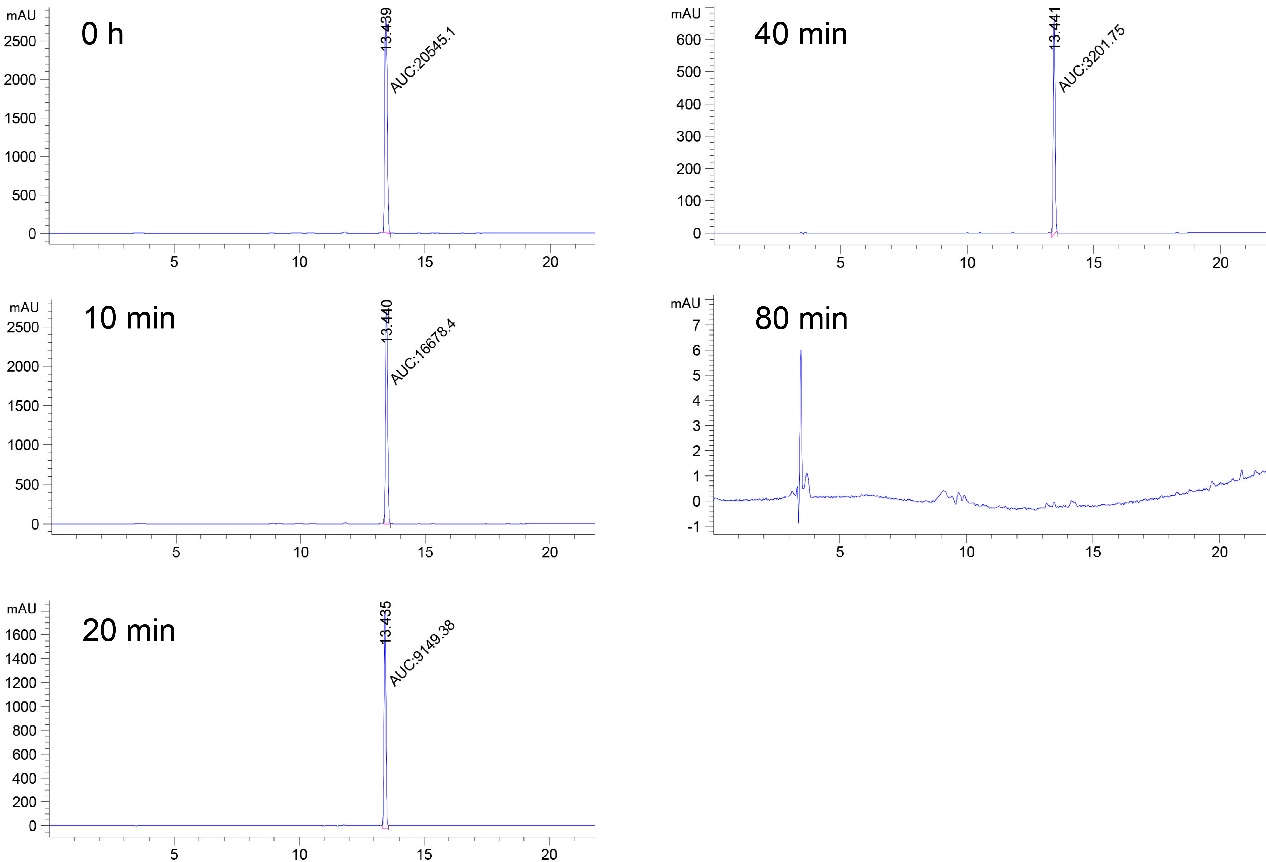


### Figure S10. Chromatograms of binding study of Acacetin-EMCH-D6 to hydroxyapatite at pH=7.4 (37 °C).


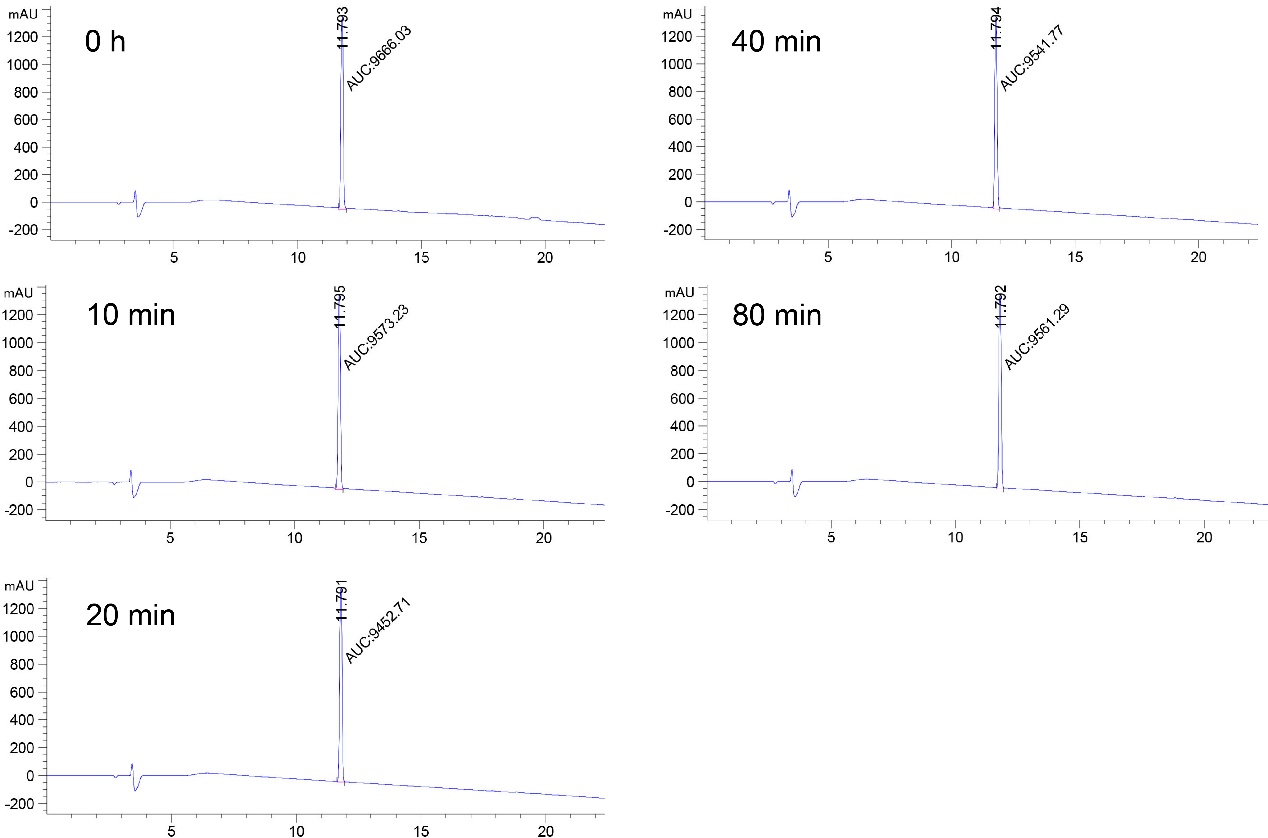


### Figure S11. Chromatograms of binding study of acacetin to hydroxyapatite at pH=7.4 (37 °C).
